# Hybrid minimally invasive correction for flexible flatfeet in young adults: a prospective cohort study

**DOI:** 10.1051/sicotj/2025070

**Published:** 2026-03-10

**Authors:** Ahmed S. Elhalawany, Ahmed Kholeif, Abo Bakr Zein, Mohamed Nagy, Mahmoud M. Elsaqa, Mohammed Ali, Ahmed Khedr

**Affiliations:** 1 Department of Orthopedics, Kasr Al Aini Faculty of Medicine, Cairo University Hospitals 11562 Cairo Egypt; 2 James Paget University Hospital A47 Lowestoft Road Gorleston-on-Sea, Great Yarmouth Norfolk NR31 6LA UK; 3 Manchester University NHS Foundation Trust Oxford Road Manchester M13 9WL UK; 4 6th Of October Hospital For Health Insurance 5 El Mosiqar Aly Ismail Street 12588 Giza Egypt; 5 University Hospital of North Durham North Rd Durham DH1 5TW UK

**Keywords:** Minimally invasive correction, Calcaneal osteotomy, Flatfoot, Arthroereisis

## Abstract

*Introduction*: This study aims to assess the functional and radiological outcomes of combining minimally invasive medial displacing calcaneal osteotomy (MDCO) with subtalar arthroereisis (STA) for the treatment of symptomatic planovalgus feet in young adults. *Methods*: A single-centre, prospective cohort study was conducted between November 2015 and February 2022. The study included a total of 32 patients with flexible flatfoot who were treated with subtalar arthroereisis combined with medialising calcaneal osteotomy with at least three years of follow-up. Radiographic evaluation included talar coverage angle, AP talo-first metatarsal (T1MT), AP talo-calcaneal, lateral talo-first metatarsal, and calcaneal pitch angles. Function was assessed by the AOFAS score. *Results*: Angles and scores were compared preoperatively and at the third-year follow-up. The mean talo-navicular coverage angle TNCA reduced from 32.72° (±8.33) preoperatively to 8.84° (±5.70) at the last follow-up. The mean AP T1MT improved from 21.59° (±8.47) preoperatively to 7.78° (±4.03) at three years postoperatively. Meary’s angle decreased from 20.84° (±7.14) preoperatively to 4.78° (±3.20) following the correction. The mean preoperative AOFAS score was 62.69 (±9.26), and significantly improved to 94.19 (±3.80) at the last follow-up. Four feet experienced sinus tarsi pain (12.5%), and three patients (9.3%) needed removal of the arthroereisis implant. *Conclusions*: The combination of MDCO and STA holds significant promise for treating flexible flatfeet in adolescents and young adults, particularly in cases of moderate to severe deformity. This combination demonstrates a synergistic interaction, with the STA implant providing internal bracing to support MDCO and reducing stresses over the medial arch by preventing hyper-pronation. Simultaneously, the MDCO reinforces the reconstruction, achieving the necessary increased correction in moderate to severe flatfoot cases, while also reducing stresses over the STA implant.

## Introduction

When treating flatfoot, it’s essential to recognise that patient and procedure selection remain a significant challenge for orthopaedic surgeons. Understanding the current difficulties in the field is crucial as we explore new techniques and approaches. This includes the growing interest in joint-preserving techniques and minimally invasive surgeries (MIS) in foot and ankle surgery, where arthroereisis is widely used in various conditions and across different age groups. By being aware of these challenges, we can better navigate the path towards improved flatfoot treatment [1–3].

Subtalar arthroereisis (STA) has been primarily used in paediatric flexible planovalgus feet, yielding satisfactory outcomes [3, 4]. The function of the hardware is to limit excessive three-dimensional movement of the subtalar joint, thereby decreasing the tendency for medial and plantar displacement of the talus and subsequently reducing stress on the medial structures. In adults, STA can be used as an adjunct to other flatfoot correction techniques [4–6]. There is still no worldwide consensus or guidance regarding its indications, age, type of patient, implant, amount of correction, limitations, pain associated with hardware, and implant removal [7, 8].

The medial displacement calcaneal osteotomy (MDCO) is used to restore the foot alignment, decrease load over the medial arch, normalise force at the talonavicular joint, reposition the Achilles tendon to function as a heel inverter, and improve patient outcomes [9].

The current study aims to assess the functional and radiological outcomes, as well as the complications, of combining minimally invasive medial displacing calcaneal osteotomy with arthroereisis for the treatment of symptomatic planovalgus feet in young adults. This study is part of an ongoing effort to expand our understanding of flatfoot correction, and further research is needed to fully explore the potential of this combined approach in this age group.

## Patients and methods

In this cohort study, the data were prospectively collected between November 2015 and February 2022 in the Foot and Ankle unit at Cairo University Hospital. It included 32 feet (26 patients) who presented with flexible flatfeet and met the criteria. The inclusion criteria included age (14–22 years old), symptomatic flexible flat feet after failure of conservative measures for more than six months, and existing foot deformity in the form of combined heel valgus, loss of the medial arch, and forefoot abduction. The exclusion criteria included previous foot operations, rigid pes planus, neurological conditions, posterior tibial tendon dysfunction, and a BMI greater than 30. All patients underwent minimally invasive medial displacing calcaneal osteotomy with subtalar arthroereisis, a procedure chosen for its proven efficacy in correcting flexible flatfeet. Patients/parents were counselled and consented to the surgical procedure and the possible complications, including sinus tarsi pain and implant removal.

After a comprehensive clinical assessment (including medical history and examination), the patients were evaluated functionally using the Orthopaedic Foot and Ankle Society (AOFAS) score [10]. This was further complemented by a rigorous radiological assessment, which was conducted using weight-bearing foot X-rays, AP and lateral views, and measurements of a range of angles. These included the AP talonavicular coverage angle (TNCA), AP talo-first metatarsal angle (T1TM), AP talocalcaneal angle (Kite’s angle), Lateral calcaneal pitch angle, and lateral talo-first metatarsal angle (Meary’s angle). The radiological evaluations were completed for all patients by two independent investigators, ensuring the highest level of accuracy and reliability in our findings.

### Surgical technique

All surgeries were performed by two senior foot and ankle orthopaedic surgeons using a standard technique. The surgery was performed in the supine position, with ipsilateral buttock support to allow access to the lateral side of the foot.

**Figure 1:**
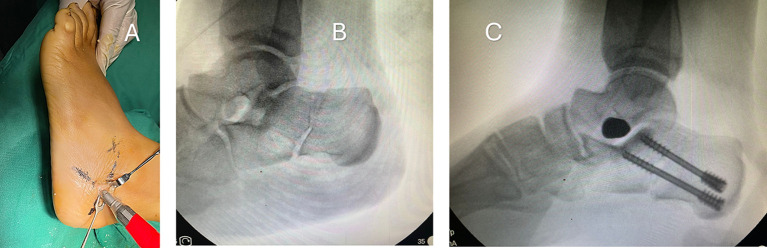
A: Intraoperative photograph of MIS MDCO using low-speed, high-torque burr. B: Intraoperative X-ray after completion of MDCO. C: MDCO after fixation with 2 screws.

The osteotomy site was marked under fluoroscopy guidance for the MDCO. A small incision (5 mm) down to the bone is made. Under fluoroscopy guidance, with saline cooling, a low-speed, high-torque burr was used to initiate the osteotomy at the near cortex. Then advanced through the calcaneus cancellous bone until it reached the far cortex, thereby preventing injury to the medial neurovascular bundle (Figure 1). The surgeon ensured that care was taken to avoid damaging the skin and surrounding soft tissues. The far cortex was carefully divided using an osteotome, and the residual medial aspect periosteum was freed to facilitate the medial displacement. Ensure that medial displacement of at least 10 mm was achieved, avoiding angulation or vertical translation of the tuberosity [11]. Once the required shift was reached, the osteotomy was stabilised using one 6.5 mm cannulated screw or double compression screw. Skin closure was then achieved using a single suture (Figure 1).

**Figure 2:**
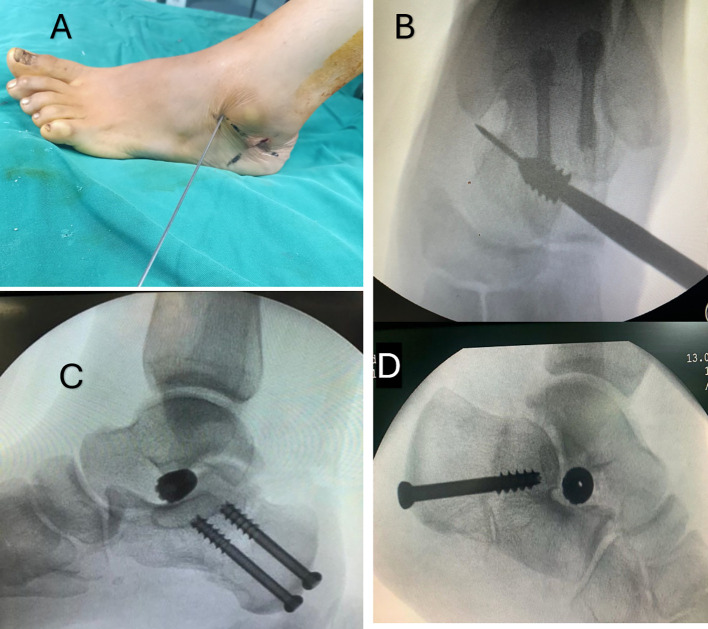
A: Intraoperative photograph of insertion of STA guidewire into tarsal canal. B: Intraoperative X-ray of insertion of STA screw over the guidewire. C,D: Final intraoperative X-ray after combination MDCO + STA in 2 different cases.

STA was performed through an incision over the sinus tarsi, which was palpated just inferior and distal to the tip of the fibula. Blunt dissection exposed the tarsal canal. A blunt guide wire was then inserted from lateral to medial, recognising that the sinus tarsi is oriented obliquely from anterolateral to posteromedial and superior. The guide wire was advanced until it tented the medial skin just dorsal to the sustentaculum tali. With the guide wire in place, the trial sizers were advanced in sequential size increments, and the range of movement and alignment of the hindfoot were assessed when each trial implant was placed. The appropriate sizer limits pathological eversion/foot pronation. If the sizer was too small, excessive eversion was present. If it was too large, the hindfoot and forefoot were overcorrected into inversion with no movement around the subtalar joint. Once the best sizer was identified, the AP radiograph was checked. On AP fluoroscopy, the trial implant or sizer typically crosses only half of the mediolateral diameter of the talar neck. It should not protrude beyond the lateral border of the talar neck. The definitive titanium conical subtalar implant was then inserted over the guide wire under image guidance, with its base being flush with the lateral border of the talar neck. Once the implant was positioned correctly, the guide wire was removed, and the wound was closed by a single skin suture (Figure 2).

Heel cord tightness was routinely checked and lengthened when necessary. In case of residual forefoot supination, a Cotton plantar flexion osteotomy of the medial cuneiform was performed through a dorsal approach and fixed with a wedge plate.

**Figure 3:**
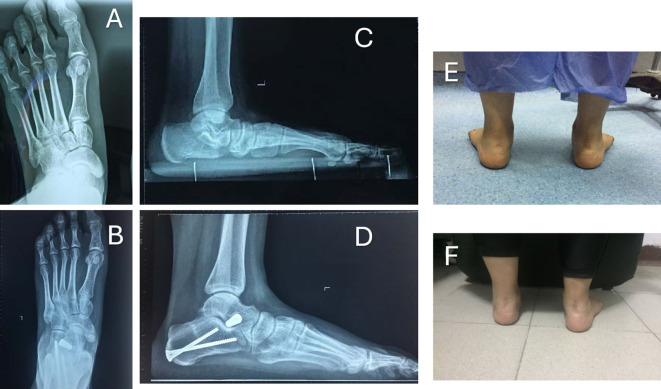
Pre- and post-operative X-rays and clinical photographs of 20-year-old female underwent left foot MDCO + STA. A: Preoperative A-P weight-bearing X-ray. B: Postoperative A-P weight-bearing X-ray. C: Preoperative lateral weight-bearing X-ray. D: Preoperative lateral weight-bearing X-ray at the last follow-up. E: Preoperative clinical photo showing the left foot hindfoot valgus. F: Postoperative clinical photo with evidence left foot hindfoot valgus correction at the last follow-up.

**Figure 4:**
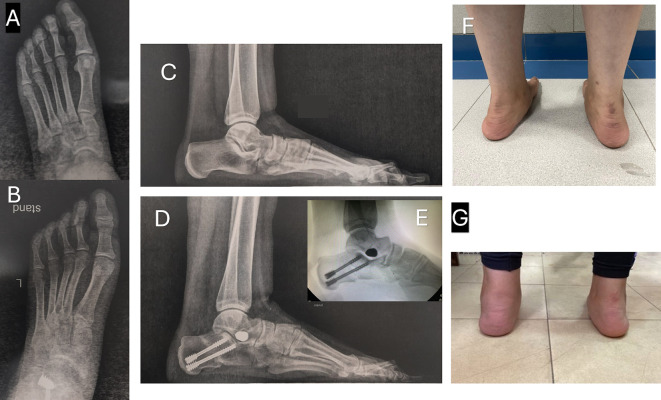
Pre- and post-operative X-rays and clinical photographs of 18-year-old female underwent left foot MDCO + STA. A: Preoperative A-P weight-bearing X-ray. B: Postoperative A-P weight-bearing X-ray. C: Preoperative lateral weight-bearing X-ray. D: Preoperative lateral weight-bearing X-ray at the last follow-up. E: Intraoperative X-ray demonstrates STA + MDCO technique. F: Preoperative clinical photo showing the left foot hindfoot valgus. G: Postoperative clinical photo with evidence left foot hindfoot valgus correction at the last follow-up.

At the end of the procedure, the incisions were covered with a sterile dressing, accompanied by the application of a cotton wool pad and a below-knee cast for six weeks. The wound was checked at two weeks, and patients continued in a below-knee cast for another four weeks. Radiographs were taken at the six-week follow-up appointment. Then the cast was removed, allowing the patient to start weight-bearing in a walker boot and begin physiotherapy rehabilitation for another six weeks. Radiographic and functional assessments were carried out at six weeks, six months, and twelve months, and then yearly (Figures 3 and 4).

## Statistical analysis

Data were coded and entered using the widely accepted statistical package for the Social Sciences (SPSS) version 28 (IBM Corp., Armonk, NY, USA). This choice of tool underlines the reliability of our study. Data were analysed using means and standard deviations for the quantitative variables and frequencies (number of cases) and relative frequencies (percentages) for the categorical variables. Comparisons between preoperative and postoperative data were performed using the paired *t*-test. *P*-values less than 0.05 were considered statistically significant*.*

## Results

A total of 41 patients were studied, of whom nine were excluded because they did not meet the inclusion criteria or were lost to follow-up. Therefore, the study included 32 feet (26 patients), 15 females (57.7%), and 11 males (42.3%). The mean age at the time of the operation was 16.53 (±2.37) years (range 14–22). The right side was affected in 13 patients, the left side was affected in 7 patients, and six patients were bilaterally affected.

Measurements and Scores were compared preoperatively and at the third-year follow-up. The mean talo-navicular coverage angle TNCA reduced from 32.72 (±8.33) preoperatively to 8.84 (±5.70) at the last follow-up (*p* < 0.001). The mean AP T1MT improved from 21.59 (±8.47) preoperatively to 7.78 (±4.03) three years postoperatively (*p* < 0.001). Meary’s angle decreased from 20.84 (±7.14) preoperatively to 4.78 (±3.20) after correction (*p* < 0.001). Kite’s angle improved from 33.69 (±7.60) before the operation to 23.34 (±4.13) at the final follow-up. Calcaneal pitch angle was changed from 10.81 (±4.79) preoperatively to 17.69 (±3.83) at the last follow-up.

The mean preoperative AOFAS score of our patients was 62.69 (±9.26), and at the last follow-up, the mean AOFAS score was 94.19 (±3.80) (*p* < 0.001) (Table 1).

**Table 1:** Comparison between the preoperative and the postoperative 3rd year follow-up scores and radiographic angles.

	Pre-operative		3 years postoperative		*P* value
	Mean	±SD	Mean	±SD	
AOFAS	62.69	±9.26	94.19	±3.80	<0.001
TNCA	32.72	±8.33	8.84	±5.70	<0.001
AP T1MT	21.59	±8.47	7.78	±4.03	<0.001
AP TCA Kite’s angle	33.69	±7.60	23.34	±4.13	<0.001
Meary’s angle	20.84	±7.14	4.78	±3.20	<0.001
Calcaneal pitch angle	10.81	±4.79	17.69	±3.83	<0.001

Adjunct procedures were done in nine patients, eleven feet (34%). These included percutaneous Achilles tendon lengthening in eight feet (25%) and cotton osteotomy in three feet (9%).

All patients in this study achieved union, all patients had restoration of neutral hindfoot alignment, all wounds healed without any noted problems, and all completed three years of follow-up with a mean length of follow-up of 39.59 (±6.68) months.

We report complications in 5 patients (5 feet). One patient had pain around the MDCO screws entry site, which improved gradually after the first three months with NSAIDs. Four patients experienced sinus tarsi pain (12.5%), one patient improved with NSAIDs, and three feet (9.3%) needed removal of STA implant at eight, nine, and twelve months from the index procedure. Following STA screw removal, the correction was maintained, and the pain resolved at the last follow-up; no further procedures were required.

None of our patients had a fractured talus, screw migration, extrusion, or degenerative changes in the subtalar joint at the final follow-up.

## Discussion

Several established operative strategies exist for managing symptomatic flexible flatfoot, including lateral column lengthening, MDCO, spring-ligament reconstruction, STA, and combined tendon procedures. Lateral column lengthening is often performed via an Evans-type osteotomy and aims to correct forefoot abduction by extending the calcaneus, thereby restoring midfoot alignment and improving load distribution. In contrast, STA relies on an implant to restrict excessive subtalar pronation without reshaping bone, making it less invasive and generally reversible. While lateral column lengthening typically provides a strong structural correction, it carries higher procedural demands, an osteotomy, and a longer recovery, whereas STA is more suitable for selected, flexible deformities that respond to subtalar motion control [12, 13]. To move toward minimally invasive techniques while maintaining satisfactory correction power, we combined minimally invasive MDCO and STA. Studies that combine these two MIS in this age group are lacking. This study is the first prospective study to combine the two minimally invasive procedures, STA and MDCO, in this age group. Our results demonstrated that STA and MDCO significantly improved the functional and radiological outcomes in patients with a symptomatic flexible flatfoot (FFF) in this age group [14, 15].

Subtalar arthroereisis can be an excellent, feasible, minimally invasive procedure for many patients with different degrees of pathology. STA provides a three-dimensional correction by hindering the talus from sliding forward, inward, and downward, thereby limiting excessive pronation. It does not affect the bone development and does not interfere with potential osteotomies that may be needed in the future [16]. However, STA alone may not be enough to correct all components of the flatfoot deformity [17].

Many studies recommend STA in adults as an adjunct rather than a standalone procedure because its correction is limited, especially in severe deformity, as this requires too much from a simple implant (Table 2) [18–23].

**Table 2:** Summary of studies on subtalar arthroereisis (STA) as adjunct to flatfoot reconstruction.

Study	Design / sample	Procedures studied	Key findings
Vora et al. [18]	Cadaveric biomechanical study	MDCO + FDL transfer ± STA	Adding STA enhanced the correction of the FFF deformity without causing any adverse biomechanical consequences. This added procedure achieved the necessary correction in the severe FFF model.
Xu Yang et al. [19]	Retrospective case series in adolescents (20 feet, age 10–14, mean FU 18.9 months).	Calcaneal Z-osteotomy + STA	STA corrected plantar-flexed talar head, improved T1MT angle. Reduced need for LCL, lowering risk of lateral column pain. Combined approach advantageous for relatively severe flexible flatfoot.
Li Bing et al. [23]	Clinical series (32 paediatric feet, age 8–12 years, mean FU 25.3 months).	STA + medial soft-tissue reconstruction	Combination effectively reconstructed FFF in children. Serve as an effective method for severe forefoot abduction reconstruction. 3.1% reoperation rate.
Walley et al. [22]	Case-control study (15 patients vs 30 controls, AAFD, mean FU 4.4 vs 3.3 years, radiographic FU 2.38 years).	MDCO + FDL + spring ligament repair + Achilles lengthening with vs without STA	Adding STA gave a better chance of achieving a normal TN coverage compared with the control group. STA improved radiographic correction. 6% sinus tarsi pain rate.
Lewis et al. [20]	Retrospective clinical study (212 feet, Stage 1 PCFD, mean FU 2.5-year follow-up).	Conventional Stage-1 PCFD procedures ± STA	Using STA as an adjunct resulted in remarkable improvements in pain and function in stage-1 flexible PCFD reconstruction. Reported high STA removal rate (48%).
Bernasconi et al. [21]	Retrospective study (22 feet, adult, Stage IIb AAFD, mean radiographic FU 11.2 months).	MDCO + FDL + spring ligament repair ± Cotton osteotomy ± STA	STA was the lone predictor of change in TNC and CFM angles. STA improved the correction of forefoot abduction. Implant-related symptom/removal rate (33%).
This Study	Prospective clinical study (32 feet, age 14–22 years old, mean FU 39.59 (±6.68) months).	MDCO + STA	MDCO + STA achieved satisfactory outcomes in young adult FFF. Sinus tarsi pain (12.5%) and STA removal (9.3%).

Abbreviations: STA: subtalar arthroereisis, FU: follow-up, FFF: flexible flatfoot, AAFD: adult-acquired flatfoot deformity, PCFD: progressive collapsing foot deformity, FDL: flexor digitorum longus transfer, MDCO: medial displacement calcaneal osteotomy, T1MT: talar-first metatarsal angle, LCL: lateral column lengthening, TNC: talonavicular Coverage angle, CFM: calcaneo–fifth metatarsal angle.

The authors chose this age group to combine these two procedures, as with physeal closure, there is a lower chance of remodelling, a more severe deformity, and subsequently a higher risk of under correction and implant-related pain in standalone arthroereisis.

A previous case-control study by Walley et al. compared MDCO, FDL transfer, spring ligament repair, and Achilles lengthening with and without STA. They concluded that patients who had additional STA had a better chance of achieving a normal talonavicular coverage compared with the control group. They also reported a six per cent risk of developing sinus tarsi pain [22].

Lewis et al. [20] retrospectively studied the functional outcomes of employing STA as an adjunct to conventional procedures in 212 feet at the first stage of flexible PCFD. Based on postoperative functional outcomes at a mean 2.5-year follow-up, they suggested that the use of STA hardware in addition to conventional procedures in the 1st stage of flexible PCFD can lead to a remarkable improvement in pain and function.

Our results are in agreement with Bernasconi et al., who retrospectively studied twenty-two feet with stage IIb adult acquired flatfoot deformity managed by MDCO, FDL transfer, spring ligament repair with or without Cotton osteotomy, and with or without STA. They found that STA was the lone predictor of change in TNC and CFM angles. STA resulted in an increased change in TNC and CFM Angles by 10.1° and 5°, respectively. Furthermore, STA improved the correction of forefoot abduction. On the other hand, four out of twelve STA patients complained of sinus tarsi pain postoperatively, and the metal removal rate was 33% [21].

Wide ranges of sinus tarsi pain (6% and 46%) and reoperation rates (9% and 39%) are described in the literature [21, 24]. Low rates of sinus tarsi pain and removal have been reported in recent studies [22, 25]. Many analyses of the risk factors associated with sinus tarsi pain have been conducted, such as patient factors, screw type, size, and position.

Regarding our complications, the main complication was pain in the sinus tarsi. Four patients (feet) experienced sinus tarsi pain (12.5%), one patient improved with NSAIDs, and three patients (9.3%) needed removal of STA. Following STA screw removal, pain was resolved, and correction was maintained at the last follow-up.

In our series, we have minimised the risk factors related to sinus tarsi pain by avoiding oversizing/ over-correction, excluding patients with a high BMI (over 30), and using STA as an adjunct, not as a standalone procedure. All our patients were counselled regarding the risk of STA pain and removal.

Our results favour the combination of both procedures; there is an exchange of benefits between the two procedures, as STA seems to aid in correcting hindfoot malalignment and corrects the medially deviated head of the talus, decreasing stresses on the medial arch and consequently protecting the osteotomy correction. On the other hand, the MDCO is considered a relatively safe and straightforward calcaneal osteotomy and could be performed utilising a minimally invasive approach and may further normalise parameters that remain under-corrected with STA and decrease the stresses over STA implant, which may be explained in our results as reduced rates of sinus tarsi pain and loss of STA screw position [9, 21].

### Study limitations

Firstly, there is no control group, thus limiting the strength of the current analysis; secondly, the small sample size is a concern. At last, the mean follow-up period of this study is 39 months, which may be relatively short for a flatfoot series. Moreover, additional adjunct procedures were not included in the analysis. Thus, the statistical findings should be interpreted with caution. Consequently, further studies are needed to elucidate the long-term outcomes of this technique. The results of this study provide a solid base for the development of future research RCTs comparing STA alone with combined procedures.

### Conclusion

The combination of the MCDO and STA could be an effective option for treating flexible flat feet in adolescents and young adults, particularly in cases of moderate to severe deformity. In contrast, either procedure alone may not be sufficient. This combination exhibits a synergistic interaction, as the STA implant internally braces the correction of MDCO and reduces stresses over the medial arch by preventing hyperpronation. At the same time, the MDCO strengthens the correction and achieves the required increased correction in moderate to severe flatfoot cases, thereby decreasing stresses over the STA implant.

## Data Availability

The data related to this study are available from the corresponding author upon reasonable request and considering any legal restrictions.
